# Hot-Tub–Associated Mycobacterial Infections in Immunosuppressed Persons

**DOI:** 10.3201/eid0807.020143

**Published:** 2002-07

**Authors:** Donald R. Graham

**Affiliations:** Springfield Clinic, Springfield, Illinois, USA

To the Editor: I read with interest the report by Mangione et al. regarding *Mycobacterium avium* infection in a Colorado family who used an inadequately sanitized hot tub (1). The authors noted that the source of the *M. avium* complex was not clear, although the reservoir did appear to have been the hot tub.

 Twenty years ago, I helped treat a patient with a local infection caused by *M. fortuitum* in his amputation stump (2). The patient had sat in his tub postoperatively three to four times per week. Although he had added disinfectants as recommended by the manufacturer, he had not cleaned the tub mechanically at any time during the incubation period of his infection. We recovered what appeared to be the same strain of *M. fortuitum* from the abscess on his amputation stump and specimens from the hot tub water and filter. However, we could not recover any Mycobacteria from his or his neighbor's tap water.

 Three years after our experience with this patient, *M. chelonei* was found to cause colonization of sputum of patients with cystic fibrosis after they had been treated in a hydrotherapy pool (3).

 These experiences indicate the absolute need for careful cleaning of hot tubs. Not only are immunosuppressed patients at risk for atypical mycobacterial infections but even otherwise healthy persons may be susceptible.
